# Association of *Interleukin-12A* rs568408 with Susceptibility to Asthma in Taiwan

**DOI:** 10.1038/s41598-017-03523-0

**Published:** 2017-06-09

**Authors:** Te-Chun Shen, Chia-Wen Tsai, Wen-Shin Chang, Shengyu Wang, Che-Yi Chao, Chieh-Lun Hsiao, Wei-Chun Chen, Te-Chun Hsia, Da-Tian Bau

**Affiliations:** 1 Graduate Institute of Clinical Medical Science, College of Medicine, China Medical University, Taichung, Taiwan, ROC; 2 Terry Fox Cancer Research Laboratory, Department of Medical Research, China Medical University Hospital, Taichung, Taiwan, ROC; 3 Division of Pulmonary and Critical Care Medicine, Department of Internal Medicine, China Medical University Hospital, Taichung, Taiwan, ROC; 4 Department of Pulmonary and Critical Care Medicine, the First Affiliated Hospital of Xi’an Medical University, Xi’an, P.R. China; 5 Department of Health and Nutrition Biotechnology, Asia University, Taichung, Taiwan, ROC; 6 Department of Respiratory Therapy, China Medical University, Taichung, Taiwan, ROC; 7 Department of Bioinformatics and Medical Engineering, Asia University, Taichung, Taiwan, ROC

## Abstract

Asthma is an inflammatory disease and interleukin 12 (IL-12) may play a regulatory role in allergen-induced inflammation. The aim of this study was to investigate the association of polymorphisms in *IL-12A/IL-12B* with asthma. The asthma group included 198 adult patients and the control group included 453 individuals without asthma that were frequency-matched by gender and age. The distribution of genotypic and allelic frequencies of *IL-12A* rs568408 demonstrated significant differences between case and control groups. Specifically, the percentages of AA genotype of *IL-12A* rs568408 was significantly higher among asthmatic patients in Taiwan than healthy controls, compared to GG genotype. No significant difference was observed among the *IL-12A* rs2243115 and *IL-12B* rs3212227 genotypes between case and control groups. In addition, the A allele at *IL-12A* rs568408 was associated with more severe symptoms (*P* = 0.0085) among asthmatic patients. These results suggest that *IL-12A* rs568408 may contribute to the etiology and symptoms severity of asthma, indicating its usefulness as a predictive and diagnostic biomarker of asthma.

## Introduction

Asthma, the most common chronic respiratory disease, is a complex disorder that demonstrates variable phenotypes and an aberrant T helper 2 (Th2) cytokine profile^1^. Asthma is characterized by reversible airflow obstruction, airway inflammation, airway remodeling, and airway hyperresponsiveness^2^. Simplistically, asthma is divided into two subtypes: allergic and non-allergic^3^. Over the past 30 years, there has been a dramatic increase in the number of patients with asthma^4^, affecting >300 million people around the world^5^. Asthma involves the interaction of genetic and environmental components. The genetic contribution to asthma susceptibility was estimated to vary between 36% and 79%^6, 7^. The understanding that genomics play a role in the pathogenesis of asthma has been recognized for >100 years^4^. The exact mechanism of asthma pathogenesis, however, is still poorly understood. Asthma is characterized as a syndrome with various unidentifiable causes underlying its development and manifestation. It is difficult to identify specific causal genes and to determine if ethnic disparities should be attributed to the genetic contribution of this complex disorder. In recent years, >200 asthma candidate genes have been proposed by different approaches, including case-control association studies and positional cloning to investigate the phenotypic functions for the positive associated variants^8, 9^.

Cytokines play a pivotal role in regulation of the immune responses and the polymorphic nature of cytokine genes may confer flexibility of cellular immune phenomenum^10^. Recent evidence suggests that deficiency in the T helper 1/2 (Th1/Th2) cytokine pathway may underlie susceptibility to asthma^11^. IL-12 is an important immune-regulatory cytokine that demonstrates an antagonistic effect on the Th1/Th2 cytokine balance and provides a functional link between innate and acquired immune responses^12^. IL-12 is derived from dendritic cells, Langerhans cells, mononuclear phagocytic cells, and mast cells. IL-12 stimulates interferon production and activates and induces proliferation and cytokine production of natural killer cells^13^. Molecularly, IL-12 is a heterodimeric cytokine composed of two disulfide-linked polypeptide chains of molecular weights 35 (p35) and 40 (p40) kDa (encoded by *IL-12A* and *IL-12B* genes, respectively). IL-12 potently induces Th1 immune responses, while promoting maturation of cytotoxic T lymphocytes, stimulation of natural killer cells, and production of interferon-gamma (IFN-γ)^14, 15^. IL-12 also impairs neo-angiogenesis in tumor tissues through induction of IFN-γ-inducible protein-10 (IP-10)^14^. Because of these pleiotropic activities, IL-12 is regarded as one of the most feasible cytokines to be applied as immunotherapy for neoplasms^16^. Because IL-12 plays such as critical role in the immune response of intercellular and intracellular microenvironments, it is plausible that *IL-12* genotypes may be determinants for individual susceptibility to human diseases, such as asthma. Previous studies have indicated that a single nucleotide polymorphism in the *IL-12B* gene (SNP; rs3212227) was significantly associated with a risk of a wide range of autoimmune and inflammatory diseases, such as psoriasis^17^, type-1 diabetes^18^, asthma-severity phenotype in Caucasians^19^, Crohn’s disease^20^, and rheumatoid arthritis^21^. Hirota and colleagues reported that the *IL-12B* rs3212227 polymorphism was significantly associated with a risk of childhood atopic asthma and was associated with high total immunoglobulin-E (IgE) levels in the Japanese population^22^. However, there are only limited studies to date that have examined the association between *IL-12B* genotypes and asthma risk, and the contribution of *IL-12A* genotypes to asthma is largely unknown. In 2011, Chen and colleagues reported that *IL-12A* rs568408 and *IL-12B* rs3212227 SNPs may individually and jointly contribute to asthma risk in a moderate population, 197 asthma patients and 369 controls in mainland China^23^. This is the first paper to examine the contribution of *IL-12* genotypes to asthma susceptibility without any further analysis of clinical features or outcomes. The Taiwanese population has a similar genetic background (Han population) to mainland China, while more Western-style culture, different pollutant exposure and dietary habits of the island country. It is important to identify genomic biomarkers of asthma in the Taiwanese population, which has a high prevalence of more than 11% of asthmatic citizens. Therefore, in the current study, *IL-12A* rs568408, *IL-12A* rs2243115, and *IL-12B rs3212227* polymorphisms were selected to investigate the associated risk of asthma in Taiwan, and their associations with clinical features, such as symptoms severity.

## Results

The distributions of demographic and clinical characteristics of the investigated population are shown in Table 1. There was no statistical difference between the two groups with respect to age and gender (*P* = 0.2739 and 0.8999, respectively). However, differences in pulmonary functions, ratio of forced expiratory volume in the first second to forced vital capacity (FEV1/FVC, %) and percent of predicted FEV1 (FEV1%), were found between the case and control groups. The percentages of asthmatic patients with 1, 2, 3 and 4 stages of symptoms severity were 30.3, 32.8, 17.2 and 19.7%, respectively (Table 1).

**Table 1:** Distribution of baseline characteristics of asthmatic patients and comparison individuals.

Characteristics	Cases (N = 198)		Controls (N = 453)		*P*-value^a^
	n	%	n	%	
Age (year)					
<37.7	98	49.5%	226	49.9%	0.9262
≥37.7	100	50.5%	227	50.1%	
mean ± SD	40.8 ± 11.8		39.7 ± 10.9		0.2739
Gender					
Men	82	41.4%	190	41.9%	0.8999
Women	116	58.6%	263	58.1%	
Pulmonary functions (mean ± SD)					
FEV1/FVC (%)	62.0 ± 13.0		80.8 ± 8.1		<0.0001
FEV1%	69.1 ± 12.9		92.9 ± 5.8		<0.0001
Symptoms severity					
1 (mild)	60	30.3%			
2	65	32.8%			
3	34	17.2%			
4 (severe)	39	19.7%			

Abbreviation: FEV1, forced expiratory volume in first second; FVC, forced vital capacity; FEV1%, percent of predicted FEV1; ^a^Chi-square without Yate’s correction test or *Student’s* t-test.

Distribution of genotypic frequencies of the three investigated SNPs, rs568408 and rs2243115 in *IL-12A* and rs3212227 in *IL-12B*, are summarized in Table 2. The Hardy-Weinberg equilibrium (HWE) *P*-values of the controls at *IL-12A* rs568408, *IL-12A* rs2243115, and *IL-12B* rs3212227 are 0.2119, 0.0001 and 0.0009, respectively. The latter two SNPs deviated from HWE, probably due to small population sizes or presence of some inbreeding. Overall, there was a significant difference in the distribution of *IL-12A* rs568408 genotypes between cases and control groups (*P* = 0.0041), but not for those of *IL-12A* rs2243115 and *IL-12B* rs3212227 (both *P* > 0.05). Specifically, frequencies of the heterozygous variant AG and homozygous variant AA of *IL-12A* rs568408 were 24.7% and 7.6% in asthmatic cases and 21.6% and 2.5% in healthy controls, respectively. The AA genotype [odds ratio (OR) = 3.50, 95% confidence interval (CI) = 1.57–7.82, *P* = 0.0022], but not AG (OR = 1.28, 95% CI = 0.86–1.91, *P* = 0.2175), at *IL-12A* rs568408 demonstrated a statistically significant risk for asthma in Taiwan. The combined variant genotypes AG + AA did not further elevate asthma risk that the OR of AA alone (1.51 versus 3.50), compared to the wild-type GG genotype, indicating that A-allele of *IL-12A* rs568408 behaves as a recessive determinant to asthma risk. After adjusted with confounding factors for asthma, age and gender, the significance and trend still exist (Table 2).

**Table 2:** Distribution of *IL-12A* rs568408, *IL-12A* rs2243115, and *IL-12B* rs3212227 genotypes among asthmatic patients and comparison individuals.

Genotype	Cases (N = 198)		Controls (N = 453)		*P-*value	OR (95%CI)	aOR (95%CI)
	n	%	n	%			
*IL-12A* rs568408							
GG	134	67.7%	344	75.9%		1.00 (reference)	1.00 (reference)
AG	49	24.7%	98	21.6%	0.2175	1.28 (0.86–1.91)	1.29 (0.87–1.92)
AA	15	7.6%	11	2.5%	0.0022*	**3.50 (1.57**–**7.82)**	**3.61 (1.60**–**8.11)**
AG + AA	64	32.3%	109	24.1%	0.0287*	**1.51 (1.04**–**2.18)**	**1.52 (1.05**–**2.19)**
*P*_*trend*_					0.0041*		
*IL-12A* rs2243115							
TT	173	87.4%	401	88.5%		1.00 (reference)	1.00 (reference)
TG	20	10.1%	45	9.9%	0.9165	1.03 (0.59–1.80)	1.03 (0.59–1.80)
GG	5	2.5%	7	1.6%	0.3947	1.66 (0.52–5.29)	1.65 (0.52–5.29)
TG + GG	25	12.6%	52	11.5%	0.6768	1.11 (0.67–1.85)	1.11 (0.67–1.85)
*P*_*trend*_					0.6899		
*IL-12B* rs3212227							
AA	73	36.9%	162	35.8%		1.00 (reference)	1.00 (reference)
AC	77	38.9%	188	41.5%	0.6250	0.91 (0.62–1.33)	091 (0.62–1.33)
CC	48	24.2%	103	22.7%	0.8810	1.03 (0.67–1.61)	1.03 (0.66–1.61)
AC + CC	125	63.1%	291	64.2%	0.7862	0.95 (0.67–1.35)	0.95 (0.67–1.35)
*P*_*trend*_					0.8137		

Abbreviations: OR, odds ratio; CI, confidence interval; aOR: adjusted OR; Model: explanatory variable = genotype (treated as categorical variable), covariable = age and gender, dependent variable = asthma; The significant *P*-value and odds ratio are bolded and marked with a star.

The distribution of allelic frequencies of rs568408 and rs2243115 in *IL-12A* and rs3212227 in *IL-12B* are summarized in Table 3. As demonstrated in Table 3, the A allele at *IL-12A* rs568408 was associated with increased risk of asthma, compared to the G allele (OR = 1.63, 95% CI = 1.19–2.23, *P* = 0.0021). Specifically, the frequencies of the A and G alleles of *IL-12A* rs568408 were 19.9 and 80.1% in asthmatic patients and 13.2 and 86.8%, respectively, in controls (Table 3). By contrast, neither the G allele of *IL-12A* rs2243115 nor the C allele of *IL-12B* rs3212227 was associated with any significantly increased risk of asthma. The adjustments of confounding factors for asthma, age and gender, did not alter the significance and trend observed (Table 3).

**Table 3:** Distribution of *IL-12A* rs568408, *IL-12A* rs2243115, and *IL-12B* rs3212227 allelic frequencies among asthmatic patients and comparison individuals.

Allele	Cases (2 N = 396)		Controls (2 N = 906)		*P-*value	OR (95%CI)	aOR (95%CI)
	n	%	n	%			
*IL-12A* rs568408							
Allele G	317	80.1%	786	86.8%		1.00 (reference)	1.00 (reference)
Allele A	79	19.9%	120	13.2%	0.0021*	**1.63 (1.19**–**2.23)**	**1.64 (1.20**–**2.25)**
*IL-12A* rs2243115							
Allele T	366	92.4%	847	93.5%		1.00 (reference)	1.00 (reference)
Allele G	30	7.6%	59	6.5%	0.4845	1.18 (0.75–1.86)	1.18 (0.74–1.86)
*IL-12B* rs3212227							
Allele A	223	56.3%	512	56.5%		1.00 (reference)	1.00 (reference)
Allele C	173	43.7%	394	43.5%	0.9469	1.00 (0.80–1.28)	1.01 (0.79–1.28)

Abbreviations: OR, odds ratio; CI, confidence interval; aOR: adjusted OR; Model: explanatory variable = allele (treated as categorical variable), covariable = age and gender, dependent variable = asthma; The significant *P*-value and odds ratio are bolded and marked with a star.

Because age and gender may play a role in asthma, we were also interested in the interaction of the *IL-12A* rs568408 genotype with age and gender of the participants. In stratified analyses by age (Table 4), it appeared that the allelic frequencies of the A allele at *IL-12A* rs568408 was significantly higher in the asthma group than the control group among people of less than 37.7 years old (*P* = 0.0007), but not among people at the age ≥37.7 years old (*P* = 0.3876). As for gender analysis (Table 4), the A allele at *IL-12A* rs568408 was significantly higher in the asthma group than the control group among women (*P* = 0.0131), but not significant among men (*P* = 0.0593). The interaction of genotype-age and genotype-gender did not reach statistical significance (P for interaction = 0.09 and 0.72), likely due to small sample sizes.

**Table 4:** Distribution of *IL-12A* rs568408 allelic frequencies among asthmatic patients and comparison individuals after stratification by age and gender.

Allele	Cases (2 N = 396)		Controls (2 N = 906)		*P-*value	OR (95% CI)	aOR (95% CI)
	n	%	n	%			
Age (year)							
<37.7							
Allele G	149	76.0	393	86.9		1.00 (reference)	1.00 (reference)
Allele A	47	24.0	59	13.1	0.0007*	**2.10 (1.37**–**3.22)**	**2.05 (1.34**–**3.15)**
≥37.7							
Allele G	168	84.0	393	86.6		1.00 (reference)	1.00 (reference)
Allele A	32	16.0	61	13.4	0.3876	1.23 (0.77–1.95)	1.28 (0.80–2.04)
*P* for interaction = 0.09							
Gender							
Men							
Allele G	126	76.8	318	83.7		1.00 (reference)	1.00 (reference)
Allele A	38	23.2	62	16.3	0.0593	1.55 (0.98–2.43)	1.47 (0.97–2.41)
Women							
Allele G	191	82.3	468	69.0		1.00 (reference)	1.00 (reference)
Allele A	41	17.7	58	11.0	0.0131*	**1.73 (1.12**–**2.67)**	**1.76 (1.14**–**2.72)**
*P* for interaction = 0.72							

Abbreviations: OR, odds ratio; CI, confidence interval; aOR: adjusted OR; Model: explanatory variable = allele (treated as categorical variable), covariable = gender, dependent variable = asthma (upper panel); explanatory variable = allele (treated as categorical variable), covariable = age, dependent variable = asthma (lower panel); The significant *P*-value and odds ratio are bolded and marked with a star.

We are interested in the association between *IL-12A* and *IL-12B* genotypes and asthma-relevant phenotypes. The distribution of allele frequencies of rs568408 and rs2243115 in *IL-12A* and rs3212227 in *IL-12B* and symptoms severity among asthmatic patients are shown in Table 5. From the data, it is found that the A allele frequencies at *IL-12A* rs568408 seemed to be lower than G allele frequencies among the patients in stage 1 and 2 (22.8 and 24.0% versus 32.2 and 35.0%, respectively), but higher than G allele frequencies among the patients in stage 3 and 4 (22.8 and 30.4% versus 15.8 and 17.0%, respectively), and the overall difference is statistically significant (*P* = 0.0085) (Table 5).

**Table 5:** Association of *IL-12A* and *IL-12B* polymorphisms with the symptoms severity among asthmatic patients.

Polymorphisms	Symptoms severity, n (%)				*P*-value^a^
	1 (mild)	2	3	4 (severe)	
*IL-12A* rs568408					
Allele G	102 (32.2)	111 (35.0)	50 (15.8)	54 (17.0)	
Allele A	18 (22.8)	19 (24.0)	18 (22.8)	24 (30.4)	0.0085*
*IL-12A* rs2243115					
Allele T	110 (30.1)	119 (32.5)	63 (17.2)	74 (20.2)	
Allele G	10 (33.3)	11 (36.7)	5 (16.7)	4 (13.3)	0.8216
*IL-12B* rs3212227					
Allele A	65 (29.1)	73 (32.7)	36 (16.2)	49 (22.0)	
Allele C	55 (31.8)	57 (32.9)	32 (18.5)	29 (16.8)	0.5970

^a^Chi-square without Yate’s correction test; The significant *P*-value and odds ratio are bolded and marked with a star.

## Discussion

In this hospital-based case-control study, we investigated the association of *IL-12A* rs568408, *IL-12A* rs2243115 and *IL-12B* rs3212227 polymorphisms and risk of asthma in Taiwan. The sample size is representative, containing 198 asthma patients and 453 age-matched and gender-matched healthy individuals (Table 1). We found that SNPs at *IL-12A* rs568408, but not *IL-12A* rs2243115 or *IL-12B* rs3212227 (Tables 2 and 3), were genomic determinants for asthma risk. In addition, the A allele at *IL-12A* rs568408 was associated with higher symptoms severity among asthmatic patients (Table 5). These findings support our hypothesis that functional polymorphisms in *IL-12* may play a role in the initiation and progression of asthma.

In the literature, genotypic and phenotypic functions of *IL-12B* rs3212227 have been extensively studied and revealed. In 2001, it was reported by Morahan and colleagues that the *IL-12B* rs3212227 AA genotype was associated with significantly elevated expression of *IL-12* among type 1 diabetes patients, with preferential transmission at this 3′-UTR polymorphism^19^. Soon after that in 2002, similar results were reported by Davoodi-Semiromi and colleagues using peripheral lymphocytes from type 1 diabetes patients in Caucasian-American families^24^. However, controversial results were reported by the following groups: In 2002, Seegers and colleagues reported that the presence of variant genotypes in *IL-12B* rs3212227 correlated with increased *in vitro* expression of *IL-12A*, but not *IL-12B* secretion^25^. In 2005, Yilmaz and colleagues found that individuals with the CC homozygous genotype at *IL-12B* rs3212227 had significantly higher IL-12 secretion levels from peripheral blood mononuclear cells stimulated by lipopolysaccharide and purified protein derivatives compared to those with AC heterozygous or AA homozygous genotypes^26^. As for cancer genomic studies, the CC/AC genotypes of *IL-12B* rs3212227 were associated with increased risk of esophageal cancer^27^, gastric cancer^28^, breast cancer^29^, bladder cancer^30^, cervical cancer^31, 32^, and osteosarcoma^33^, but some controversial findings were also found^34–37^. These inconsistencies may come from different use of inclusive and exclusive criteria during sample collection and different populations investigated. In addition, stimulation protocols and immunological status of patients were different. In our results, there was no association between the genotypes of *IL-12B* rs3212227 and asthma risk (Tables 2 and 3). Chen and colleagues reported that the AC genotype of *IL-12B* rs3212227 was associated with a borderline decreased risk of asthma compared with the AA genotype (*P* = 0.036)^23^. The subjects investigated were very similar between the Chen study and our study (controls versus cases = 369/197 versus 453/198). The percentage of AA, AC and CC genotypes was 36%, 49.1% and 14.9% in the Chen control population, and 35.8%, 41.5% and 22.7% in our control population.

In the current study, we identified a molecular risk biomarker, the AA genotype at *IL-12A* rs568408, for asthma susceptibility in early detection and prediction. This genotype was associated with elevated risk in several types of cancer, including esophageal cancer^27^, colorectal cancer^37^, liver cancer^35^, cervical cancer^31^, and osteosarcoma^33^. *IL-12B* can interact with *IL-12A*, in charge of formation of IL-12, IL-23A, and subsequent IL-23. Thus, the complicated and dynamic interaction among immune-proteins may play a very critical role in controlling immunoreactions in extracellular microenvironments. Thus, it is worthwhile for scientists to investigate the gene-gene interaction between *IL-12A* and *IL-12B* and their possibility to be novel determinants for asthma susceptibility, as is the case for cancer genomic etiology. In 2009, Chen and colleagues reported that *IL-12A* rs568408 GA/AA and *IL-12B* rs3212227 AC/CC variant genotypes were associated with a significantly increased risk of cervical cancer^31^. They proposed that the three SNPs in this study are located in conserved regions in mice, such that rs3212227 may disrupt exonic splicing silencers, and that rs568408 may disrupt exonic splicing enhancers and miRNAs binding^31^. Another novel finding in the current study was that the A allele at *IL-12A* rs568408 was associated with more severe symptoms among asthmatic patients (Table 5). However, the expression levels of IL-12 in investigated subjects were not available, which therefore limited our further analysis of the genotype-phenotype correlation and the possible association of phenotypic characteristics with prognosis and outcomes. In the near future, our findings could be validated in other asthma populations to determine if the AA *IL-12A* rs568408 genotype is a common genomic biomarker for asthma in these populations. Chen and his colleagues reported that the AG genotype of *IL-12A rs568408* was associated with an increased risk of asthma compared to the GG genotype (*P* < 0.001)^23^, while they did not find any people with AA genotype and analyze the association of *IL-12* genotypes with clinical features. Compared with their findings, the sample size of our study is of the same level (controls versus cases = 369/197 versus 453/198). In detail, percentages of GG, AG and AA genotypes was 80.2%, 19.8% and 0% in their control population, and 75.9%, 21.6% and 2.5% in ours. The lack of AA genotype at *IL-12A rs568408* in the Chen population is unusual and was not observed in previous studies of other diseases performed in other areas of China^31, 37, 38^ or by the recording on National Center for Biotechnology Information website (https://www.ncbi.nlm.nih.gov/projects/SNP/snp_ref.cgi?rs = 3212227). The inconsistency may be related to differences in criteria of inclusion and exclusion during sample selection and collection.

There were some limitations in the current study. First, several asthma-related clinical information and phenotypes were not well recorded, such as the therapeutic responsiveness after bronchodilators and IgE levels. The restrictiveness limited us for further analysis about the role of *IL-12* in asthma-related clinical outcomes. Second, the limited sample size may prone to lead false positive or negative findings due to lower statistical analyzing power. Third, the environmental factors such as occupational and allergen exposing status, which may serve as important confounding factors for asthma, were not well self-recalled and recorded.

## Conclusion

The current study found that A allele at *IL-12A rs568408* may act as a recessive determinant to asthma etiology and symptoms severity. We believe that our work may contribute to the understanding for the role of IL-12 in precise prediction, prevention and personalized strategies for asthma. Further investigations on the phenotypes of A allele at *IL-12A rs568408* are needed.

## Methods

### Study population

A total of 198 patients with asthma were recruited at China Medical University Hospital. The medical history was reviewed and recorded into the database. At the same time, 453 healthy individuals, who had been frequency-matched with asthma patients by gender and age (±5 years) and who had no previous history of asthma, were admitted to the same hospital for health screening (similar residential areas) and enrolled as matched controls. All individuals enrolled were provided with informed consent and the study was evaluated and approved by the Research Ethics Committee of China Medical University and Hospital (DMR100-IRB-284). All methods were performed in accordance with the relevant guidelines. The diagnosis and severity for each asthma patient were verified and agreed by at least two experienced pulmonary specialists according to the Global Initiative for Asthma (GINA) guidelines [The Global Strategy for Asthma Management and Prevention. Global Initiative for Asthma (GINA) 2010. Available from: http://www.ginasthma.org]. Based on the clinical features presented, the patients were further categorized into four severity stages by the pulmonary specialists. The stage 1 is mild and stage 4 is severe. After the interview, 5 ml of venous blood was collected from each individual and used for DNA extraction and genotyping assays.

### Genotyping conditions

Genomic DNA from peripheral blood leukocytes of patients and comparison individuals was prepared using the QIAamp Blood Mini Kit (Blossom, Taipei, Taiwan) and further processed as in our previous article^39^.

Primer sequences were designed as published by Chen and colleagues^31^. For *IL-12A* rs2243115 and rs568408, a mismatched A to replace C and a mismatched T to replace C were introduced into forward primers, respectively, at −3 bp from the polymorphic sites to create *Bsen I* and *Nde I* (New England BioLabs, Ipswich, MA, USA) digestible restriction sites. The primers were 5′-AGAAAAGACCTGTGAACAAAACGACT-3′ (forward) and 5′-AGATGGCTCACTAGATGCCAGG-3′ (reverse) for *IL-12A* rs2243115, and 5′-GAAGGATGGGACYATTACATCCATAT-3′ (forward) and 5′-CAGGATGGATATTTTCCCTTCT-3′ (reverse) for *IL-12A* rs568408. The variant allele *IL-12A* rs2243115G produced two fragments of 93 and 29 bp and the wild-type allele *IL-12A* rs2243115T generated one fragment of 122 bp. As for *IL-12A* rs568408, the wild-type allele *IL-12A* rs568408G produced 2 fragments of 98 and 23 bp and the variant allele *IL-12A* rs568408A resulted in one fragment of 121 bp. The primers of *IL-12B* rs3212227 were 5′-GATATCTTTGCTGTATTTGTATAGTT-3′ (forward) and 5′-AATATTTAAATAGCATGAAGGC-3′ (reverse), which generated a 118-bp fragment. Fragments were then digested by *Taq I* (New England BioLabs, Ipswich, MA, USA). The variant allele *IL-12B* rs3212227C produced 2 fragments of 92 and 26 bp, and the wild-type allele *IL-12B* rs3212227A resulted in a single 118-bp fragment.

Polymerase chain reaction (PCR) cycling conditions were performed as follows: one cycle at 94 °C for 5 min; 35 cycles of 94 °C for 30 s, 55 °C for 30 s, and 72 °C for 30 s; and a final extension at 72 °C for 10 min. Products were separated on a 3% agarose gel at 100 volts for 20 min. Genotype analysis was performed blinded by three independent researchers. Approximately 5% of the samples for each SNP were randomly selected for direct sequencing and the results from PCR-RFLP and direct sequencing were 100% concordant.

### Statistical analyses

All of the 198 cases and 453 controls containing genotypic and clinical data were analyzed. To ensure that controls were representative of the general population and to exclude the possibility of genotyping error, the deviation of the genotype frequencies of *IL-12* SNPs in the control subjects from those expected under the HWE was assessed using the goodness-of-fit test. *Student’s t*-test was used for comparison of average age and pulmonary functions between case and control group. Pearson’s chi-square test was used to compare the distribution of age group, gender, and *IL-12* genotypes/SNP alleles between cases and controls. Logistic regression was used to estimate crude and adjusted ORs with 95% CIs. Any *P* < 0.05 was considered statistically significant.
